# Lateral–Medial Dissociation in Orbitofrontal Cortex–Hypothalamus Connectivity

**DOI:** 10.3389/fnhum.2016.00244

**Published:** 2016-05-26

**Authors:** Satoshi Hirose, Takahiro Osada, Akitoshi Ogawa, Masaki Tanaka, Hiroyuki Wada, Yasunori Yoshizawa, Yoshio Imai, Toru Machida, Masaaki Akahane, Ichiro Shirouzu, Seiki Konishi

**Affiliations:** ^1^Department of Neurophysiology, Juntendo University School of MedicineTokyo, Japan; ^2^Department of Physiology, The University of Tokyo School of MedicineTokyo, Japan; ^3^Department of Radiology, NTT Medical Center TokyoTokyo, Japan; ^4^International University of Health and WelfareTokyo, Japan

**Keywords:** functional MRI, neuroanatomy, brain mapping, frontal lobe, orbitofrontal cortex

## Abstract

The orbitofrontal cortex (OFC) is involved in cognitive functions, and is also closely related to autonomic functions. The OFC is densely connected with the hypothalamus, a heterogeneous structure controlling autonomic functions that can be divided into two major parts: the lateral and the medial. Resting-state functional connectivity has allowed us to parcellate the cerebral cortex into putative functional areas based on the changes in the spatial pattern of connectivity in the cerebral cortex when a seed point is moved from one voxel to another. In the present high spatial-resolution fMRI study, we investigate the connectivity-based organization of the OFC with reference to the hypothalamus. The OFC was parcellated using resting-state functional connectivity in an individual subject approach, and then the functional connectivity was examined between the parcellated areas in the OFC and the lateral/medial hypothalamus. We found a functional double dissociation in the OFC: the lateral OFC (the lateral orbital gyrus) was more likely connected with the lateral hypothalamus, whereas the medial OFC (the medial orbital and rectal gyri) was more likely connected with the medial hypothalamus. These results demonstrate the fundamental heterogeneity of the OFC, and suggest a potential neural basis of the OFC–hypothalamic functional interaction.

## Introduction

Although the orbitofrontal cortex (OFC) has been well-documented in relation to cognitive functions (Mishkin, 1964; Robbins, 1996; Kringelbach and Rolls, 2004; O’Doherty, 2004; Schoenbaum et al., 2009; Rushworth et al., 2011), its involvement in autonomic functions has been less highlighted. Electric stimulation to the OFC leads to autonomic responses such as changes in blood pressure, heart rate, and respiration rate (Kaada et al., 1949). The OFC is connected, both directly and indirectly, with the hypothalamus (Ongur and Price, 2000; Barbas, 2007). The hypothalamus is recognized as the highest-level center of autonomic functions, and synthesizes the sympathetic/parasympathetic nervous system and the endocrine system (Kandel et al., 2013). The hypothalamus is a small structure (approximately 1 cm^3^ per hemisphere in humans) that contains several nuclei, and can conventionally be divided into two parts: the lateral and the medial. The lateral part roughly corresponds to one relatively large area, the lateral hypothalamic area, and is known as the hunger center (Anand and Brobeck, 1951; Delgado and Anand, 1953). The medial part of the hypothalamus contains several nuclei, including the ventromedial nucleus known as the satiety center (Hetherington and Ranson, 1940; Miller, 1960), and is related to various autonomic functions such as cardiovascular regulation, hormonal release, and circadian rhythms (Kandel et al., 2013). Thus the OFC may be involved in autonomic functions via its connections with the hypothalamus. Although the OFC–hypothalamus connections have been well described in animal studies (Ongur et al., 1998; Rempel-Clower and Barbas, 1998), the precise anatomical architecture in humans is not well understood.

Functional MRI has allowed us to reveal connectivity-based areal dissociations among small brain structures such as the locus coeruleus in comparison with the ventral tegmental area/substantia nigra pars compacta (Zhang et al., 2016) and the basal nucleus of Meynert in comparison with the ventral striatum (Li et al., 2014). Moreover, areal parcellation using clustering methods has been developed using diffusion-weighted imaging (Behrens et al., 2003; Johansen-Berg et al., 2004; Anwander et al., 2007; Klein et al., 2007; Mars et al., 2011) and resting-state functional connectivity (Yeo et al., 2011; Kahnt et al., 2012; Zhang et al., 2012b; Hutchison et al., 2013, 2015; Onoda and Yamaguchi, 2013; Shen et al., 2013; Long et al., 2014; Eickhoff et al., 2015; Finn et al., 2015; Poldrack et al., 2015; Wang et al., 2015; Reid et al., 2016). In particular, Kahnt et al. (2012) revealed six functionally distinct regions in the OFC using clustering method in resting-state functional connectivity. More recently, resting-state functional connectivity has allowed us to map boundaries of putative functional areas (Margulies et al., 2007; Cohen et al., 2008; Biswal et al., 2010; Nelson et al., 2010; Hirose et al., 2012, 2013; Milham, 2012; Wig et al., 2014a,b; Gordon et al., 2016). For the hypothalamus, despite pioneering attempts to parcellate the human hypothalamus using diffusion-weighted imaging (Schindler et al., 2013; Schönknecht et al., 2013), it is difficult to parcellate the human hypothalamus into individual sub-nuclei with the spatial resolution provided by the current MRI technique.

**FIGURE 1:**
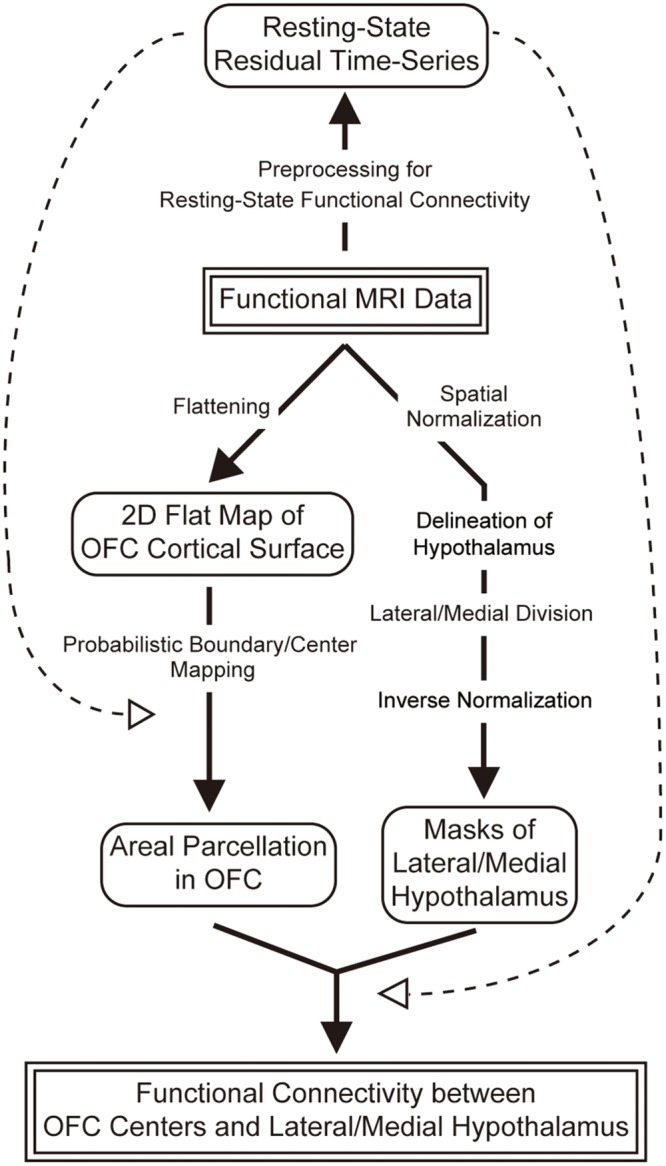
**An overview of image data analysis in the present study.** Functional MRI images were processed for (1) areal parcellation of the OFC, (2) division of the hypothalamus into the lateral and medial, and (3) calculation of functional connectivity between each of the parcellated areas in the OFC and the lateral/medial hypothalamus.

As one of early steps toward understanding the human OFC–hypothalamic interaction, in the present study, we investigated the connectivity-based architecture of the OFC by applying resting-state functional MRI with high spatial resolution of 2 mm cubic voxels (Hirose et al., 2012, 2013) in an individual-subject approach (Laumann et al., 2015; Wang et al., 2015). The OFC was parcellated into modular areas using the boundary mapping method, and the resting-state functional connectivity, which is known to primarily reflect anatomic connections (Buckner et al., 2008), was then examined between the parcellated OFC areas and the lateral/medial hypothalamus, a conventionally common separation of the hypothalamus (**Figure 1**).

## Materials and Methods

### Subjects and MRI Procedures

Twelve healthy right-handed subjects (aged 20–39 years; seven males and five females) participated in the experiments after they gave written informed consent in accordance with the Declaration of Helsinki. The procedures of fMRI experiments were approved by the Institutional Review Board of The University of Tokyo School of Medicine. The experiments were conducted using a 3T scanner (Philips Achieva X 3T Rel. 2.6, Best, The Netherlands). T1-weighted structural images were collected (resolution = 0.81 mm × 0.81 mm × 1.20 mm). Functional imaging was conducted by using gradient-echo echo-planar sequences (TR = 9.0 s, TE = 35 ms, flip angle = 90 deg, FOV = 192 mm × 192 mm, matrix size = 96 × 96, resolution = 2.0 mm × 2.0 mm × 2.0 mm, 75 contiguous transverse slices, ascending interleaved order). The data were sampled using the cubic voxels of 2 mm to minimize signal contamination from the other bank of a sulcus. The higher spatial resolution required a TR of 9 s, but the long TR will not influence the outcome because signals in a lower frequency range of the temporal filter (0.009–0.08 Hz; Fox et al., 2005; Fair et al., 2007), which are known to be predominant in functional connectivity (Salvador et al., 2005), were spared for the resting-state fMRI analysis. During the functional imaging, the subjects were instructed to passively view a fixation point on the screen. One run took about 5 min (35 volumes after discarding the first three volumes), and each subject underwent 90 runs. Typically, one session consisted of 15 runs, and two sessions were administered in 1 day, and subjects underwent these sessions in three separate days.

**FIGURE 2:**
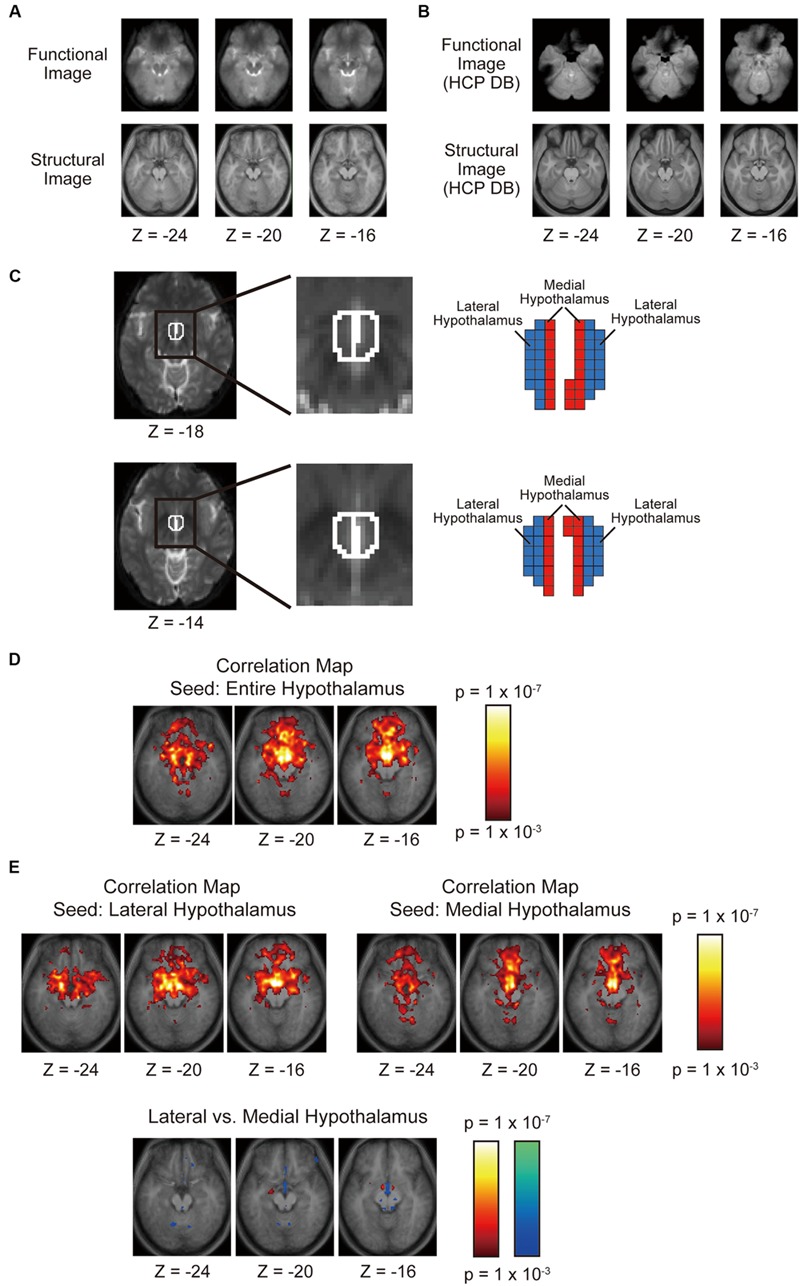
**(A)** Horizontal sections of group-averaged functional and structural images of the OFC used in the present study. **(B)** Horizontal sections of group-averaged functional and structural images of the OFC taken from the data platform in the Human Connectome Project. **(C)** The left and right hypothalami delineated using anatomical landmarks. The hypothalamus was divided into the lateral and medial parts such that their volumes of were matched with one another. **(D)** Voxel-wise statistical maps of correlation with the whole left and right hypothalami overlaid on top of the anatomic images. The color scale indicates the correlation value. **(E)** Voxel-wise statistical maps of correlation with the lateral or the medial hypothalamus. The format is similar to that in **(D)**.

**Figure 2A** shows group-averaged functional images of the OFC used in the present study. In the MNI space, the OFC ranged approximately from *Z* = -30 to -10 and *Y* = 20–60 (Chiavaras et al., 2001). Despite moderate signal loss due to the sinuses, the orbital surface of the functional images taken in this MRI system appeared relatively well-preserved. As a reference, **Figure 2B** shows group-averaged functional images of twenty subjects randomly sampled from the data platform of Human Connectome Project^1^. Although the data platform provides a standard large sample of high-resolution functional images, the large signal dropout centered around the medial OFC in either one hemisphere is less suitable for the boundary mapping analysis employed in the present study.

### Analysis Overview

An overview of the image data analysis of the present study is shown in **Figure 1**. Acquired functional images were used for (1) areal parcellation in the OFC, (2) delineation of the hypothalamus and its division into the lateral and medial parts, and (3) calculation of functional connectivity between the OFC and the hypothalamus. More specifically, the OFC was parcellated into functional areas, and their centers were defined as regions of interest (ROIs) in the OFC. The hypothalamus was defined based on anatomical landmarks, and was divided into the lateral and medial parts such that their volumes were matched with one another. The functional connectivity was then calculated between each of the centers of the parcellated areas in the OFC and the lateral/medial hypothalamus.

### Preprocessing for Resting-State Functional Connectivity

Functional images were preprocessed for the resting-state functional connectivity analysis, and were used later for areal parcellation in the OFC and for calculation of functional connectivity between the OFC and the hypothalamus. Images were realigned and were slice-timing corrected using SPM8 (Worsley and Friston, 1995). Neither spatial normalization nor spatial smoothing was used in order to maintain the higher spatial resolution of the functional images. The following preprocessing procedures are essentially the same as those used in previous literatures of resting-state functional MRI (Fox et al., 2005; Fair et al., 2007). Temporal filters (0.009 Hz < f < 0.08 Hz) were applied to the functional images using FSL (Smith et al., 2004). A general linear model (Worsley and Friston, 1995; Miezin et al., 2000) was used to regress out nuisance signals that correlated with head motion, whole-brain global signal, averaged ventricular signal, and averaged white matter signal (Fox et al., 2005; Fair et al., 2007). Both the global and white matter signals were removed together as covariates of no-interest, and the correlation between the two signals will not affect the results. To examine the effect of global signal regression on the results, the left and right OFC of the representative subject was analyzed without the global signal regression (**Supplementary Figures S1** and **S2**). The results confirmed that the global signal regression had little effect on the analyses of the present study. To avoid phase shifting, we employed frame-wise displacement (FD; Power et al., 2012, 2014) and evaluated the amount of head motion. The FD is instantaneous head motion that can be calculated as locational difference between two successive images in a run. We excluded those runs from analysis where large FD (>0.6 mm) occurred by more than 10% of images in the runs. There were 79 ± 16 runs (mean ± SD) per subject included in the analysis, and the FD was 0.18 ± 0.05 mm (mean ± SD). These runs were concatenated in subsequent correlation analyses.

### Areal Parcellation

The probabilistic boundary maps were generated based on the boundary mapping method (Cohen et al., 2008; Nelson et al., 2010; Hirose et al., 2012, 2013; Gordon et al., 2016). The OFC was flattened into the 2D space based on a cortical surface-based analysis (Fischl et al., 1999) using Caret (Van Essen et al., 2001). Each pixel in the 2D flattened cortical space was used as the seed for calculation of correlation with the target voxels. The target voxels were restricted to only those located in the corresponding region in the contralateral hemisphere (Hirose et al., 2012, 2013), without calculation of correlations with all the voxels in the whole brain in the original parcellation method. This modification of the original method has been validated by the observation that a region has the strongest functional connectivity with the corresponding region in the contralateral hemisphere (Stark et al., 2008). It has also been shown that the modified method is efficient in a signal to noise ratio, with a minimal calculation time (Hirose et al., 2012). More specifically, for each seed voxel in the OFC, a spherical region (radius: 2 mm) was generated in the contralateral hemisphere, and the spherical regions were ‘or’-combined across the seed voxels, to form a collection of target voxels. A voxel-wise correlation map in the target region was generated for each seed voxel, and the correlation coefficient was then converted to the Fisher’s *z* (Fox et al., 2005; Fair et al., 2007).

The analysis procedures after correlation map generation are basically the same as those described in the previous studies (Cohen et al., 2008; Nelson et al., 2010). It is assumed that the pixels at which the spatial pattern of the correlation maps changes drastically represent the boundaries between functional areas. The changes of the spatial pattern of the correlation maps were quantified using the similarity (index of eta^2^) of the correlation maps between the seeds. The Canny edge detection method (Canny, 1986) was used for the eta^2^ maps to create a gradient map and also to detect edges. Averaging across the entire sets of binary edge maps generated a probabilistic boundary map where intensity of the pixel represents the probability of the pixel being an edge. The local minima in the gradient map were also detected, and the binary local minimum maps were generated. The binary maps were then averaged to generate a probabilistic ‘center’ map.

We next estimated the overall distance between the centers of the adjacent parcellated areas. Since the probabilistic center maps exhibited a spatially periodic pattern of probability, an autocorrelation analysis was applied to the probabilistic center maps (Hirose et al., 2013). The 2D autocorrelation was calculated in the probabilistic center map using the Signal Processing Toolbox of MATLAB, after spatial smoothing (FWHM: 4 mm). A sliding window of a 50 mm × 50 mm space was moved across the probabilistic center map of OFC (70 mm × 70 mm). The autocorrelation was computed repeatedly for each window position and was averaged across the positions. The autocorrelation value was further clumped along the same radial coordinates to neglect directions.

### Hypothalamus Division

The masks that delineated the hypothalamus of individual subjects were created in MNI space after spatial normalization of functional images, based on anatomical landmarks in structural and functional images described in previous MRI studies of the hypothalamus (Schindler et al., 2013; Schönknecht et al., 2013; see Table 1 in Schönknecht et al., 2013 for the detail of definition). The hypothalamus masks were then divided into the lateral and medial parts by a parasagittal plane in the MNI space (**Figure 2C**). Ideally, the hypothalamus should be divided based on anatomical landmarks, but there are very few anatomical landmarks that can be seen inside the hypothalamus in structural and functional images. So the hypothalamus was split into two parts by a parasagittal plane such that the volumes of the lateral and medial parts of the hypothalamus were matched. Matching volumes helps to minimize the effect of their volumes on the significance level of functional connectivity with the OFC.

The hypothalamus mask in the MNI space created for each subject was converted back to the original individual brain to calculate the functional connectivity in the original subject space. The volume of the hypothalamus in the original subject space was 754 ± 121 mm^3^ and 779 ± 124 mm^3^ (mean ± SD) in the left and right hypothalamus, respectively, and was consistent with those reported in the previous studies (Schindler et al., 2013; Schönknecht et al., 2013; Gabery et al., 2015).

### Orbitofrontal–Hypothalamic Interaction

The functional connectivity was calculated between each of the parcellated areas in the OFC and the lateral and medial parts of the hypothalamus. The spherical ROIs (radius: 3 mm) were placed on the centers of the parcellated areas in the OFC. The significance level for functional connectivity was set at *P* < 0.05 for each parcellated area, Bonferroni corrected by the number of centers in the OFC for each subject/hemisphere (35.8 ± 7.0 and 32.9 ± 6.8, mean ± SD, in the left and right OFC).

To investigate the connectivity pattern of many parcellated areas in the OFC with the hypothalamus, the orbital surface of the prefrontal cortex in each subject was sectioned into five parts (the lateral orbital gyrus, the anterior orbital gyrus, the posterior orbital gyrus, the medial orbital gyrus, and the rectal gyrus), based on the sulcus landmarks (the lateral orbital sulcus, the medial orbital sulcus, the transverse orbital sulcus and the olfactory sulcus; Chiavaras and Petrides, 2000; Rolls et al., 2015). The positions of the sulci were provided by the sulcal depth maps in Caret software.

To investigate the connectivity pattern in the OFC in another way, the orbital surface was divided into the lateral and medial parts in a simple geometrical manner. The boundaries surrounding the orbital surface were first delineated, and the three vertices in the orbital surface were determined as the anterior vertex, the lateral posterior vertex, and the medial posterior vertex. The orbital surface was divided into the lateral and the medial parts by the line connecting the anterior vertex and the middle point of the line connecting the lateral posterior and the medial posterior vertices.

As a standard analysis of functional connectivity, a voxel-wise second-level analysis of Fisher’s *z* maps was separately conducted (**Figures 2D,E**). After preprocessing including spatial normalization to the standard template and spatial smoothing (FWHM: 6 mm), functional connectivity was calculated between the lateral/medial part of the hypothalamus and each of the voxels in the whole brain, and was converted to Fisher’s *z*. The resultant correlation *z* images for individual subjects were entered into a second-level group analysis using a random effect model.

## Results

As a standard analysis, the functional connectivity was calculated between the bilateral entire hypothalamus and each voxel in the whole brain. The group analysis showed correlation in the OFC, and major correlation clusters were located in the medial part of the OFC (**Figure 2D**). The hypothalamus was divided into the lateral and medial parts, and these parts of the hypothalamus were used as separate seed regions. The OFC showed correlation with both the lateral and medial parts of the hypothalamus primarily in the medial part of the OFC, but the difference in the correlation between the lateral and the medial hypothalamus was detected only in (6, 42, -28; medial > lateral, *t* = 5.4; **Figure 2E**).

**FIGURE 3:**
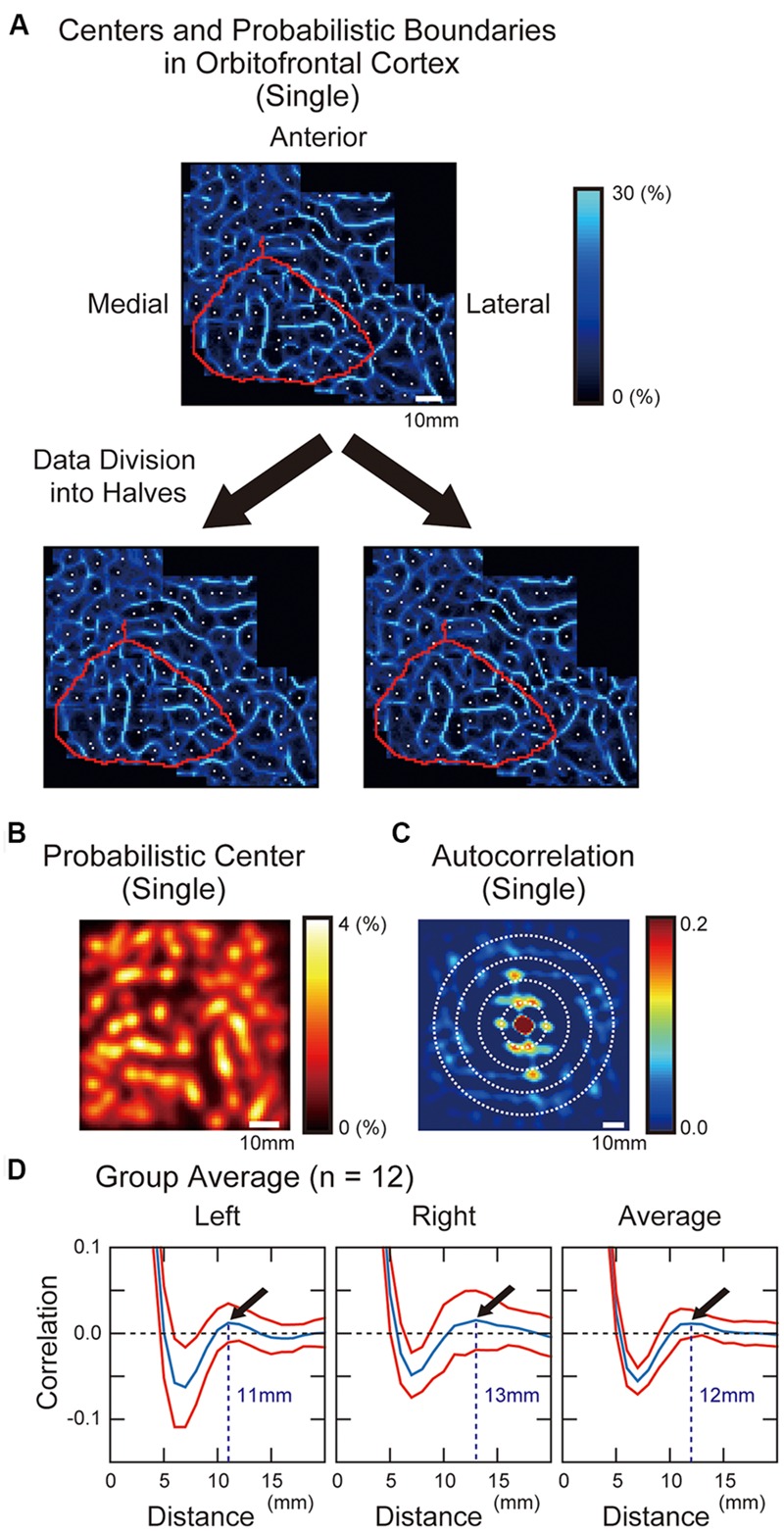
**(A)** Probabilistic boundary and center maps of the OFC generated by the areal parcellation method. Probabilistic boundaries are shown in blue, with a brightness scale that reflects probability of positive binary edges. White dots indicate the centers of the parcellated areas. Purple curve indicates anatomical landmarks surrounding the orbital surface of the prefrontal cortex. The boundary pattern was preserved even when the whole images were divided into two halves. **(B)** A probabilistic center map of the OFC. The color scale reflects the probability of local minima in the gradient maps. **(C)** Autocorrelogram of the probabilistic center maps of the OFC. To measure the overall distance between adjacent centers of the parcellated areas, periodicity of the center map was quantified using an autocorrelation analysis. The color scale indicates the correlation values at particular spatial lags. **(D)** The correlation values averaged along circles with different radii in the autocorrelogram. Red curves indicate standard error of means of the 12 subjects.

Areal boundaries and centers were then calculated, in an individual subject approach. **Figure 3A** shows a modular pattern of parcellated areas in the OFC in one representative subject. To confirm the reliability of the areal parcellation, we divided the whole image data in one representative subject into two halves, and each half was applied to the areal parcellation method. The observed pattern was successfully replicated across the two halves of data (**Figure 3A**). We next measured the overall average distance between adjacent parcellated areas based on the autocorrelation analysis. The probabilistic center map (**Figure 3B**) was used to calculate the autocorrelation (**Figure 3C**), and the autocorrelogram was averaged along circles of different radii (**Figure 3D**). The local maxima was observed at the radius of 11 and 13 mm in the left and right hemispheres respectively (average: 12 mm), indicating that the overall distance between the centers of the parcellated areas was similar to that observed in the posterior inferior frontal cortex (12 mm) reported previously (Hirose et al., 2013).

**FIGURE 4:**
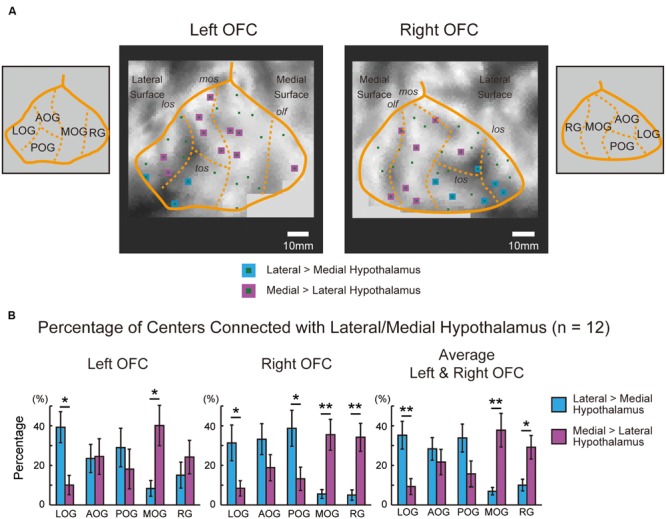
**(A)** Five parts of the OFC in one representative subject that were sectioned using sulcus landmarks. The sections were delineated in the 2D space using the lateral orbital sulcus, the medial orbital sulcus, the transverse orbital sulcus, and the olfactory sulcus, based on Chiavaras and Petrides (2000) and Rolls et al. (2015). The sulcal depth map of Caret in the representative subject was shown in a gray scale. The areal centers that were correlated significantly more with the lateral than the medial hypothalamus are shown in blue, and those was correlated significantly more with the medial than the lateral hypothalamus are shown in purple. LOG, lateral orbital gyrus; AOG, anterior orbital gyrus; POG, posterior orbital gyrus; MOG, medial orbital gyrus; RG, rectal gyrus; los, lateral orbital sulcus; mos, medial orbital sulcus; tos, transverse orbital sulcus; olf, olfactory sulcus. **(B)** Percentages of areal centers in the left and right OFC that were connected differentially with the lateral and medial hypothalamus. The percentages were group averaged across the 12 subjects in each of the five sections. The bars in the graphs indicate the standard errors of means. ^∗^*P* < 0.05, ^∗∗^*P* < 0.01.

To analyze the functional connectivity pattern of parcellated areas in the OFC with the hypothalamus, the orbital surface was sectioned into five parts in the 2D space based on sulcus landmarks (Chiavaras and Petrides, 2000; Rolls et al., 2015). **Figure 4A** demonstrates the functional connectivity in the OFC centers in one representative subject that were correlated significantly more with the lateral than the medial hypothalamus, or were correlated significantly more with the medial than the lateral hypothalamus. **Table 1** shows the summary of the number of the areas in the five OFC parts that were correlated differentially more with the lateral or the medial hypothalamus in all 12 subjects. The group data of the left and right OFC (**Figure 4B**) showed that the areas in the lateral orbital gyrus were more likely connected with the lateral hypothalamus, whereas the areas in the medial orbital gyrus and the rectal gyrus were more likely connected with the medial hypothalamus [the lateral orbital gyrus: lateral > medial, *t*(11) = 3.7, *P* < 0.01; the medial orbital gyrus: medial > lateral, *t*(11) = 3.2, *P* < 0.01; the rectal gyrus: medial > lateral, *t*(11) = 2.7, *P* < 0.05]. The anterior and posterior orbital gyri, located in-between the lateral and medial orbital gyri, did not show the differential pattern.

**Table 1:** The number of areal centers in the sulcus-based OFC sections that were connected differentially with the lateral or the medial hypothalamus.

	LOG			AOG			POG			MOG			RG		
	Total	L HT > M HT	M HT > L HT	Total	L HT > M HT	M HT > L HT	Total	L HT > M HT	M HT > L HT	Total	L HT > M HT	M HT > L HT	Total	L HT > M HT	M HT > L HT
**Left OFC**														
Case 1	8	1	2	4	0	4	3	2	0	12	0	3	2	0	1
Case 2	8	1	1	8	0	2	5	0	4	8	0	7	5	0	2
Case 3	10	5	0	6	1	0	2	0	0	8	0	1	4	0	2
Case 4	4	1	0	7	0	3	6	0	0	8	0	4	6	0	3
Case 5	4	1	0	8	2	0	1	0	1	10	4	0	9	2	0
Case 6	4	2	0	9	1	2	7	3	0	7	2	0	4	1	0
Case 7	4	2	0	9	4	0	3	0	0	19	4	1	6	1	1
Case 8	3	0	0	4	0	0	6	3	0	12	0	2	4	0	0
Case 9	2	1	1	7	5	2	4	4	0	23	1	20	6	4	0
Case 10	2	2	0	8	2	5	7	3	2	26	0	23	6	0	5
Case 11	8	5	0	8	5	1	11	5	1	17	1	12	2	1	0
Case 12	6	2	2	8	2	0	6	0	0	13	0	5	2	0	0
**Right OFC**														
Case 1	6	2	0	7	1	2	7	3	0	8	0	3	4	0	2
Case 2	4	0	0	6	0	3	4	0	2	11	0	3	6	0	2
Case 3	2	0	0	9	5	1	5	2	0	15	2	3	5	1	2
Case 4	6	3	0	4	1	0	6	0	0	15	1	7	5	1	0
Case 5	2	0	0	6	1	0	4	3	0	6	0	0	2	0	0
Case 6	6	4	0	12	1	0	11	4	0	12	0	0	2	0	0
Case 7	3	0	0	4	2	0	2	0	0	13	0	2	8	0	2
Case 8	2	0	0	10	3	0	5	1	0	11	0	2	5	0	3
Case 9	6	2	2	5	1	3	2	1	1	16	3	11	6	0	4
Case 10	5	2	1	6	3	2	3	2	1	14	0	12	5	1	3
Case 11	7	6	1	7	2	3	12	4	3	13	1	6	4	0	1
Case 12	3	2	1	4	4	0	2	2	0	10	2	6	4	0	2

*OFC, orbitofrontal cortex; LOG, lateral orbital gyrus; AOG, anterior orbital gyrus; POG, posterior orbital gyrus; MOG, medial orbital gyrus; RG, rectal gyrus; HT, hypothalamus; L, lateral; M, medial.*

**FIGURE 5:**
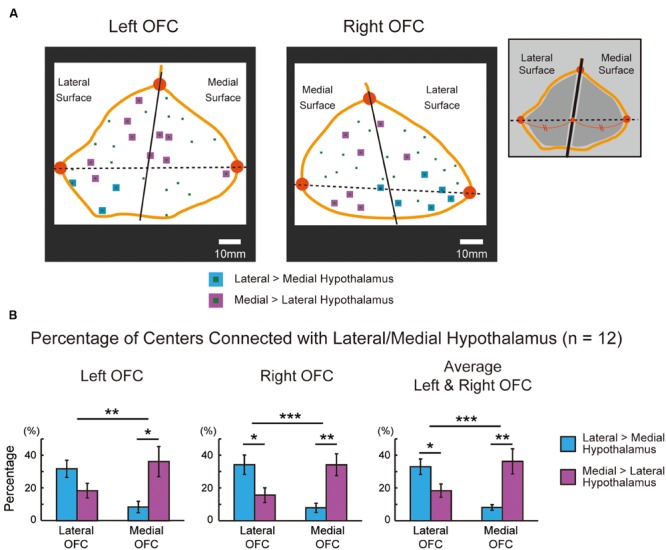
**(A)** The lateral and medial parts of the orbital surface that were sectioned geometrically in the same representative subject. The sections were delineated in the 2D space using three vertices shown in orange (anterior, lateral posterior, and medial posterior). The format is similar to that in **Figure 4A**. **(B)** Percentages of areal centers in the left and right OFC that were connected differentially with the lateral and medial hypothalamus. The percentages were group averaged across the 12 subjects in the lateral and medial OFC. The format is similar to that in **Figure 4B**. ^∗^*P* < 0.05, ^∗∗^*P* < 0.01, ^∗∗∗^*P* < 0.001.

The functional connectivity pattern in the OFC was analyzed further by sectioning the orbital surface geometrically into the lateral and medial parts in the 2D space. **Figure 5A** demonstrates the functional connectivity in the OFC centers in the same representative subject, similarly to **Figure 4A**. **Table 2** shows the summary of the number of the areal centers in the lateral and medial parts of the OFC. The group data of the left and right OFC (**Figure 5B**) showed that the lateral OFC contained the greater number of areas with greater connectivity with the lateral hypothalamus, whereas the medial OFC contained the greater number of areas with greater connectivity with the medial hypothalamus [the lateral OFC: lateral > medial, *t*(11) = 2.7, *P* < 0.05; the medial OFC: medial > lateral, *t*(11) = 3.1, *P* < 0.01]. The interaction in a two-way ANOVA with the lateral/medial OFC and the lateral/medial hypothalamus as main effects was also significant [*F*(1,11) = 34.0, *P* < 0.001].

**Table 2:** The number of areal centers in the geometry-based OFC sections that were connected differentially with the lateral or the medial hypothalamus.

	Left OFC						Right OFC					
	Lateral OFC			Medial OFC			Lateral OFC			Medial OFC		
	Total	L HT > M HT	M HT > L HT	Total	L HT > M HT	M HT > L HT	Total	L HT > M HT	M HT > L HT	Total	L HT > M HT	M HT > L HT
Case 1	16	3	6	13	0	4	18	5	2	14	1	5
Case 2	20	1	7	14	0	9	11	0	3	20	0	7
Case 3	13	5	0	17	1	3	20	7	2	16	3	4
Case 4	14	1	2	17	0	8	19	4	2	17	2	5
Case 5	13	3	1	19	6	0	11	4	0	9	0	0
Case 6	16	5	2	15	4	0	19	8	0	24	1	0
Case 7	14	5	1	27	6	2	9	2	0	21	0	4
Case 8	12	3	0	17	0	2	19	4	1	14	0	4
Case 9	15	10	5	27	0	23	12	3	6	23	4	15
Case 10	20	7	9	29	0	26	15	7	5	18	1	14
Case 11	25	14	2	21	3	12	26	13	6	17	0	8
Case 12	10	4	2	25	0	6	6	5	1	17	5	8

## Discussion

The areal parcellation method applied to high-resolution resting-state functional MRI data of individual human subjects demonstrated a basic anatomical architecture of the OFC–hypothalamus interaction: the lateral OFC (the lateral orbital gyrus) was more likely connected with the lateral hypothalamus, whereas the medial OFC (the medial orbital gyrus and rectal gyrus) was more likely connected with the medial hypothalamus. The results provide an important step toward understanding the functional interaction between the OFC and the hypothalamus.

Since the hypothalamus is a small structure of approximately 1 cm^3^ (Schindler et al., 2013; Schönknecht et al., 2013; Gabery et al., 2015), it is difficult to parcellate the hypothalamus into its individual sub-nuclei. The present resting-state fMRI study only reports the lateral–medial dissociation in the OFC–hypothalamus functional connectivity based on the lateral–medial division of the hypothalamus. Since the resting-state functional connectivity primarily reflects anatomical connections (Buckner et al., 2008), the present study also suggests underlying anatomic connectivity between the OFC and hypothalamus. The indirect connections via the third region may drive the functional connectivity, but it has been demonstrated that the majority of functional connectivity is derived from the direct connections (Honey et al., 2009; Adachi et al., 2012). Moreover, the lateral–medial dissociation is consistent with previous anatomic tracer studies of macaque monkeys (Ongur et al., 1998; Rempel-Clower and Barbas, 1998). These studies revealed differential connection patterns between the lateral hypothalamus and the OFC, and between the medial hypothalamus and the medial prefrontal cortex. Although the present study revealed a dissociation within the OFC, the orbital to medial prefrontal trend that goes along the lateral to medial hypothalamus of the monkeys is consistent with the present dissociation pattern of the lateral to medial OFC trend.

A standard group analysis revealed functional connectivity with the lateral or the medial hypothalamus primarily in the medial OFC (**Figure 2E**), consistent with the previous study of OFC parcellation (Kahnt et al., 2012). The standard group analysis did not reveal a double dissociation of the lateral–medial differential connectivity (**Figure 2E**), presumably due to larger individual variations of anatomical architecture in the association cortex (Fischl et al., 2008). One recent study has successfully reported that a right medial OFC region is connected differentially more with the medial than lateral hypothalamus (Kullmann et al., 2014), consistent with the results of the present study (**Figure 2E**). The effectiveness of the boundary mapping method in defining ROIs with homogeneous connectivity patterns (Gordon et al., 2016) may have helped reveal the lateral–medial double dissociation of the OFC–hypothalamus connectivity in the present study. It is important to discriminate signals of one bank of a sulcus from those of the other bank, and cubic voxels of 2 mm seem sufficient for this purpose. The size of the parcellated areas in the OFC shown in the present study was approximately 12 mm, and the 2 mm voxels also seem sufficient for the parcellation pattern. To examine smaller structures, however, smaller voxels will be useful, but with the need to collect a greater amount of data to compensate for lower signal to noise ratio. Future studies would be required with higher spatial resolution to parcellate the hypothalamus further, as has been done in previous studies of larger subcortical structures such as the thalamus (Behrens et al., 2003), the amygdala (Bzdok et al., 2012), and the striatum (Choi et al., 2012; Janssen et al., 2015).

The lateral–medial dissociation of the OFC–hypothalamus connectivity may explain some of autonomic aspects of the OFC in terms of hypothalamic functions. For example, the somatic marker (physiological arousal) that supports decision making, as measured with skin conductance response (Bechara et al., 2000; Zhang et al., 2012a, 2014, 2015), might be provided from the medial hypothalamus, such as the dorsomedial and the anterior nuclei. These nuclei regulate the sympathetic nervous system, and may explain the loss of skin conductance response following lesions to the OFC (Bechara et al., 2000). Impaired extinction following selective lesions to the medial OFC (Butter, 1969) might be caused by excessive desire for food reward, after loss of interaction with the ventromedial nucleus of the hypothalamus, the satiety center (Hetherington and Ranson, 1940; Miller, 1960). Further understanding of precise anatomical architecture of the OFC–hypothalamus connectivity may reveal some aspects in cognitive tasks that have largely been ignored in neuroscience literatures.

## Author Contributions

SH, TO, and SK designed the study, HW, YY, YI, TM, MA, IS, and SK collected data, SH, TO, AO, MT, and SK analyzed the data, SH, TO, and SK wrote the manuscript.

## Conflict of Interest Statement

The authors declare that the research was conducted in the absence of any commercial or financial relationships that could be construed as a potential conflict of interest.

## Footnotes

1 http://www.humanconnectome.org/data/

## Supplementary Material

FIGURE S1 **(A)** Probabilistic boundary and center maps with global signal regression (GSR). The right OFC of case 1 is shown. The format is similar to that of **Figure 3A**. **(B)** Similar to **(A)** but without GSR. **(C)** The correlation values averaged along circles with different radii in the autocorrelogram with GSR. The format is similar to that of **Figure 3D**. **(D)** Similar to **(C)** but without GSR. **(E)** Five parts of the OFC that were sectioned using sulcus landmarks. Correlation with the lateral/medial hypothalamus was calculated with GSR. The format is similar to that of **Figure 4A**. **(F)** Similar to **(E)** but without GSR. **(G)** The lateral and medial parts of the orbital surface that were sectioned geometrically. The format is similar to that of **Figure 5A**. **(H)** Similar to **(G)** but without GSR.

FIGURE S2 **The same analyses in **Supplementary Figure S1** were performed on the left OFC of case 1.** The format is similar to that of **Supplementary Figure S1**.
