# Supplementary figures and images for: Lateral–Medial Dissociation in Orbitofrontal Cortex–Hypothalamus Connectivity
Source: Front Hum Neurosci. 2016 May 26;10:244. doi: 10.3389/fnhum.2016.00244 (PMC4880561; doi:10.3389/fnhum.2016.00244)

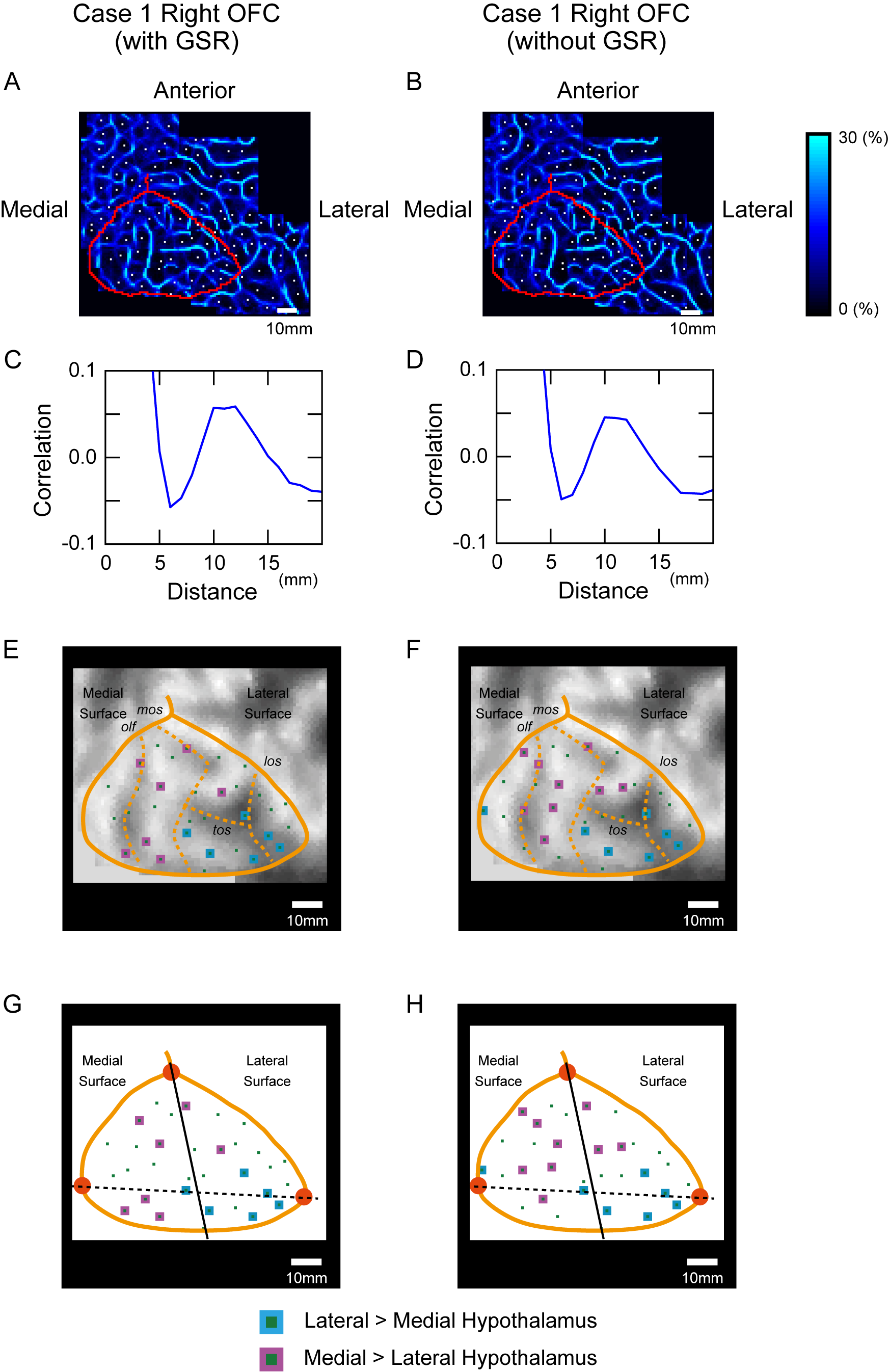

Supplement: FIGURE S1 — (A) Probabilistic boundary and center maps with global signal regression (GSR). The right OFC of case 1 is shown. The format is similar to that of Figure 3A. (B) Similar to (A) but without GSR. (C) The correlation values averaged along circles with different radii in the autocorrelogram with GSR. The format is similar to that of Figure 3D. (D) Similar to (C) but without GSR. (E) Five parts of the OFC that were sectioned using sulcus landmarks. Correlation with the lateral/medial hypothalamus was calculated with GSR. The format is similar to that of Figure 4A. (F) Similar to (E) but without GSR. (G) The lateral and medial parts of the orbital surface that were sectioned geometrically. The format is similar to that of Figure 5A. (H) Similar to (G) but without GSR. [file Image_1.TIF]

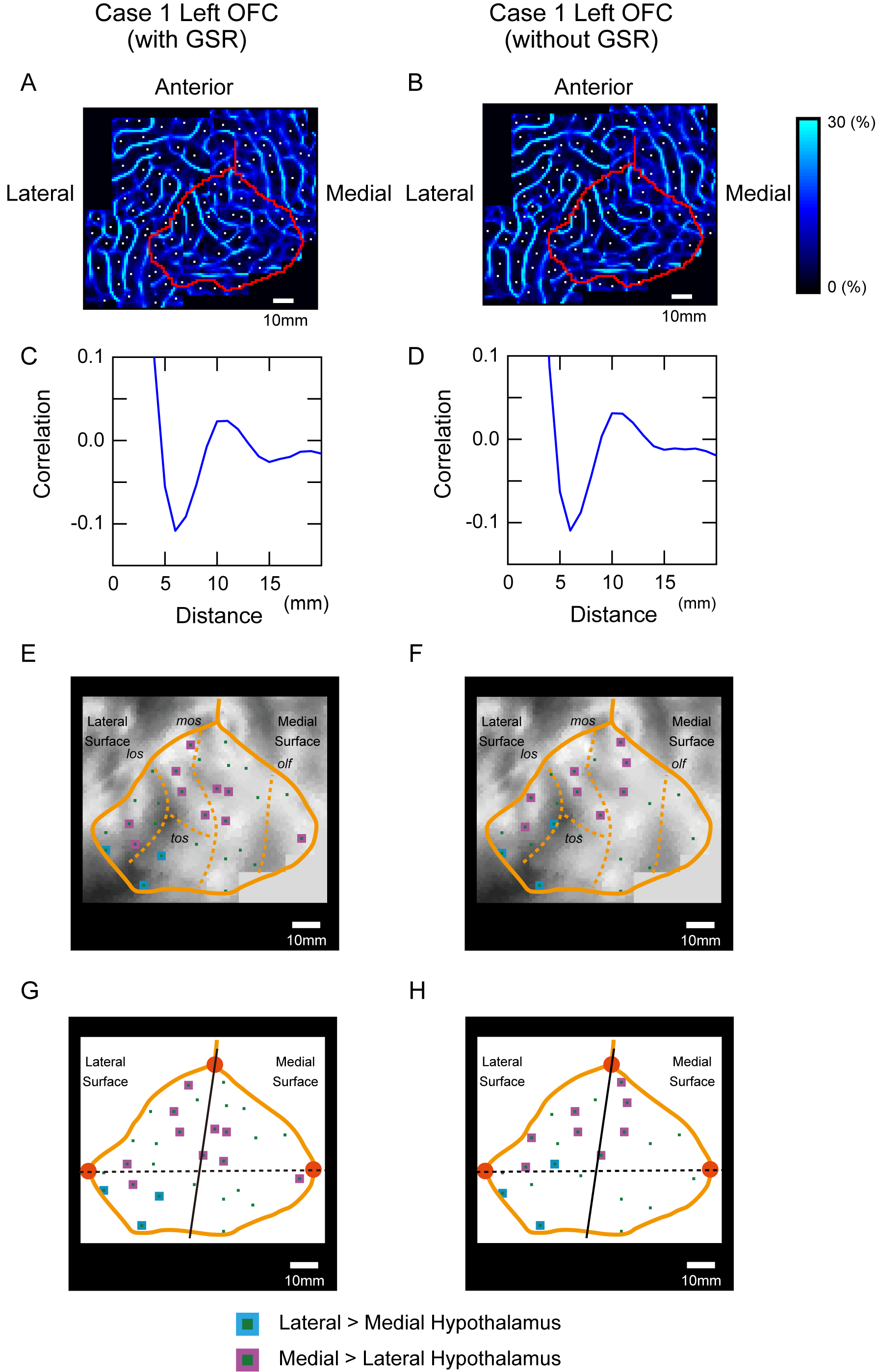

Supplement: FIGURE S2 — The same analyses in Supplementary Figure S1 were performed on the left OFC of case 1. The format is similar to that of Supplementary Figure S1. [file Image_2.TIF]
